# Elevated contextual fear memory by SIRT6 depletion in excitatory neurons of mouse forebrain

**DOI:** 10.1186/s13041-018-0391-6

**Published:** 2018-09-06

**Authors:** Hyopil Kim, Hyun-Seok Kim, Bong-Kiun Kaang

**Affiliations:** 1 Laboratory of Neurobiology, School of Biological Sciences, Seoul National University, Seoul, 08826 South Korea; 2 The Research Center for Cellular Homeostasis, Ewha Womans University, Seoul, 03760 South Korea

**Keywords:** Sirtuin, SIRT6, Behavior, Learning, Spatial memory, Contextual fear memory

## Abstract

A class of NAD-dependent protein deacetylases, the Sirtuin (SIRT) family of proteins is involved in aging, cell survival, and neurodegeneration. Recently, SIRT proteins, including SIRT6, have been reported to be important in learning and memory. However, the role of SIRT6 in excitatory brain neurons in cognitive behaviors is not well characterized. We investigated how cognitive behaviors are affected by genetic SIRT6 depletion in excitatory neurons in the mouse forebrain. We generated a conditional knockout (cKO) mouse line by mating two transgenic lines, Floxed SIRT6 and CaMKIIa-Cre. SIRT6 was thus deleted by Cre recombinase in CaMKIIa-expressing excitatory neurons. We performed cognitive behavioral tests, focusing on learning and memory, including contextual fear conditioning and Morris-water maze. The freezing level of SIRT6 cKO before the fear conditioning was comparable to that of wild-type littermate controls, while the freezing level after the conditioning was higher in SIRT6 cKO mice. In contrast, the mice showed normal spatial learning and memory in the Morris-water maze. In addition, anxiety and locomotion were also normal in SIRT6 cKO mice. SIRT6 genetic depletion enhanced contextual fear memory without affecting spatial memory. Since a previous report showed that overexpression of SIRT6 reduced contextual fear memory, our results suggest that the expression level of SIRT6 bi-directionally regulates contextual fear memory in mice.

**Electronic supplementary material:**

The online version of this article (10.1186/s13041-018-0391-6) contains supplementary material, which is available to authorized users.

## Main text

Sirtuins (SIRTs) are a class of nicotinamide adenine dinucleotide (NAD)-dependent deacetylases that have been found to be involved in aging and cellular stress in various species [1–3]. There are seven mammalian SIRT proteins with varying localizations and functions. Among the SIRT proteins, SIRT6 is prominently localized in the nucleus and its insufficiency promotes genomic instability and aging [3, 4]. Furthermore, SIRT6 plays neuroprotective roles, preventing neurodegenerative events [5]. SIRT6 deacetylates histone H3 lysine 9 (H3K9) and this modulates the protective roles [6]. Since epigenetic regulations such as DNA methylation and histone acetylation also mediate learning and memory, SIRT6 would be involved in the processes [7, 8].

Supporting this, SIRT proteins have been implicated in learning and memory. For instance, mice deficient in SIRT1, another SIRT localized in the nucleus as well as the cytoplasm, show deficits in memory and synaptic plasticity such as long-term potentiation [9]. Insufficiency of SIRT3, which is expressed in mitochondria and involved in neuroprotection, results in deteriorated remote memory [10]. The loss of SIRT6 in neuronal progenitors resulted in the accumulation of toxic tau proteins and severe deficits in both associative and non-associative memory [5]. Therefore, SIRT6 is thought to be important for learning and memory. Interestingly, however, overexpression of SIRT6 also impaired long-term contextual fear memory [11].

To further examine the relationship between SIRT6 and learning and memory, we generated a conditional SIRT6 knockout (SIRT6 cKO) by mating Floxed SIRT6 mice with mice expressing Cre recombinase under the control of the Ca^2+^/calmodulin-dependent kinase IIa (CaMKIIa) promoter. CaMKIIa is a marker of excitatory neurons of the forebrain; hence, CaMKIIa-promoter-driven Cre deletes SIRT6 in those neurons. Mice with a genotype of SIRT6 fl/fl; CaMKIIa-Cre +/− were used as SIRT6 cKO since SIRT6 can be deleted in Cre expressing neurons. Littermate mice with a genotype of SIRT6 fl/fl; CaMKIIa-Cre −/− were used as WT controls (Additional file 1: Figure S1). All the experimental procedures including animal cares are presented in the Additional file 2.

**Fig. 1:**
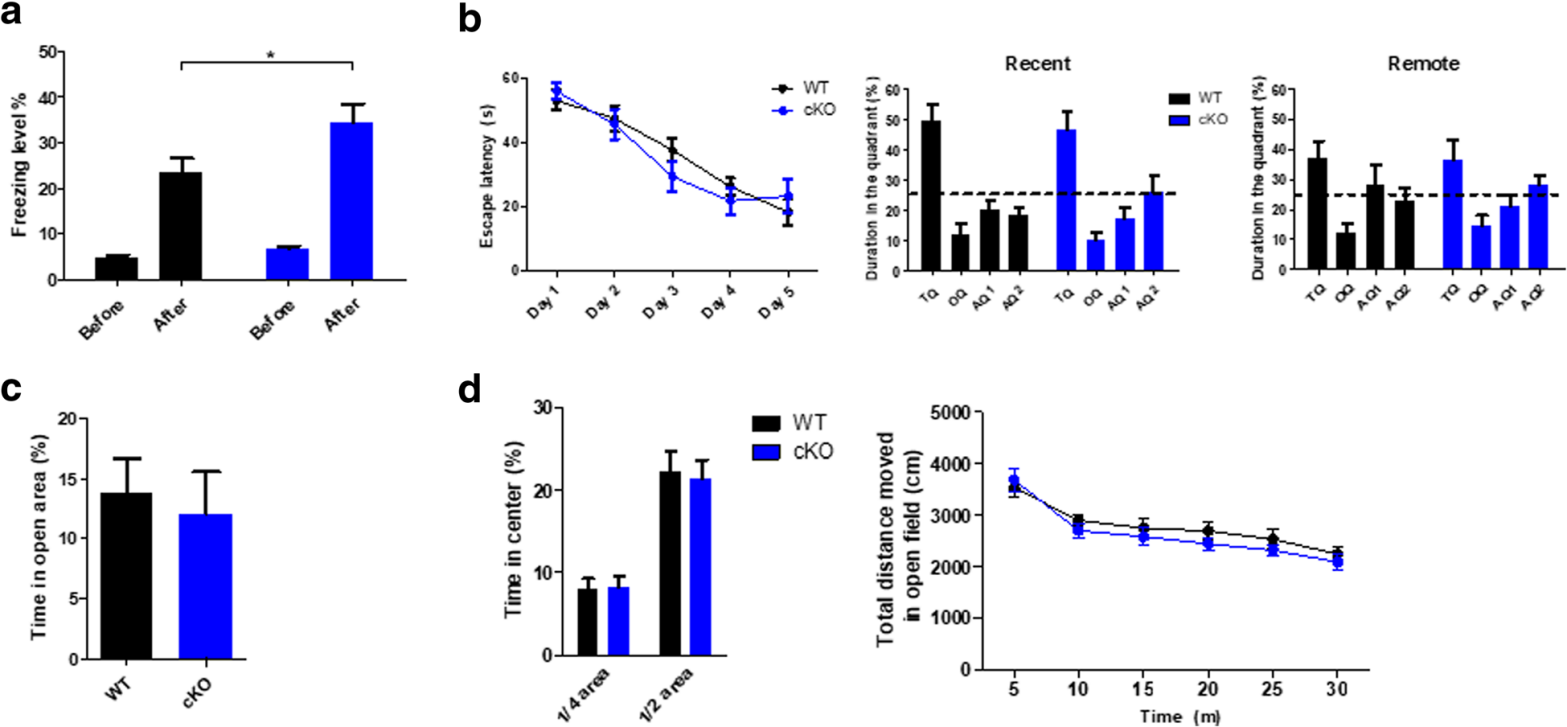
Sirtuin 6 (SIRT6) genetic inactivation in excitatory forebrain neurons enhances contextual fear memory without affecting spatial memory, anxiety and locomotion in mice. **a** Fractions of time spent freezing (freezing levels) before and 1 day after conditioning in a contextual fear conditioning test (*n* = 20 for WT mice, and *n* = 19 for SIRT6 cKO mice). **b** Left, latency to find the platform in a Morris water maze during the training period. Middle, Proportions of time spent in each quadrant in a probe test 1 day after training. Right, proportions of time spent in each quadrant in a probe test 28 days after training (*n* = 8 per group). **c** Time spent in the open segments of an elevated zero maze, a measure of anxiety (*n* = 8 per group). **d** Left, time spent in the center of the open field box, another measure of anxiety. Right, distance traveled in each 5-min interval during the open field test (*n* = 8 per group). All graphs show means ± SEM. *, *P* < 0.05 (Student’s *t*-test)

At first, we examined contextual fear memory in SIRT6 cKO mice. We assessed the percentage of the time spent freezing (freezing levels) before and 1 day after shock conditioning. While the freezing levels before shock conditioning were comparable between SIRT cKO and control mice, the post-conditioning freezing level of the cKO mice was higher than that of the WT mice (Fig. 1a, Student’s *t*-test and two-way ANOVA; genotype x shock, *p* < 0.05). There was no significant interaction in the two-way ANOVA test, but the effect of genotype was significant.

In addition, we performed a Morris-water maze test to assess spatial learning and memory. In the training session, the escape latency of cKO mice on day 3 was tended to be lower than that of WT, but the difference was not significant and the latency value was too high, so we trained the mice two more days. The learning curve of the cKO mice during the 5 days of training was comparable to the WT controls, as was their memory in a probe test performed 1 day after the final training day (Recent; Fig. 1b, left and middle, Additional file 3: Figure S2). We performed another probe test 28 days post-training (Remote) because SIRT3 KO mice were reported to have a remote memory deficit. However, remote memory in SIRT6 cKO mice was also comparable to that of WT mice (Fig. 1b, right, Additional file 3: Figure S2).

Finally, we assessed anxiety and locomotive behaviors since the factors can affect various behaviors such as the freezing level. SIRT6 cKO mice showed comparable levels of anxiety in both elevated zero maze and open field tests (Fig. 1c, d). Locomotion in SIRT6 cKO mice also was not significantly different from that in WT mice (Fig. 1d).

In the present study, we assessed the effect of genetic SIRT6 depletion in excitatory neurons on behaviors related to learning and memory. Contextual fear memory was elevated by SIRT6 depletion, contrary to a previous report showing memory impairment following genetic SIRT6 inactivation in neuronal progenitors [5]. However, since the other group targeted total neuronal populations rather than excitatory neurons, the discrepancy may be attributable to SIRT6 depletion in inhibitory neurons resulting in memory impairments. Furthermore, because another report [11] showed that SIRT6 overexpression in the CA1 region of the hippocampus impaired contextual fear memory, the memory enhancement observed in our study may indicate specific involvement of SIRT6 function in excitatory CA1 neurons in memory processes in mice. Moreover, because SIRT6 overexpression impaired the Insulin like growth factor (IGF)/Akt signaling pathway, which activates cAMP response element-binding protein (CREB), this pathway may be activated and contribute to the contextual fear memory enhancement in SIRT6 cKO mice [11, 12].

Interestingly, unlike contextual fear memory, spatial memory was not affected in SIRT6 cKO mice. Dysregulation of conditioned fear responses are involved in post-traumatic stress disorder (PTSD), hence the selective enhancement of negative memory of SIRT6 cKO mice suggests that reduced SIRT6 activity may be implicated in the disorder. However, in relation with the spatial memory, the possibility of over-training in the Morris-water maze test or another type of spatial memory tests, such as 8 arm maze test can be examined.

## Additional files

Additional file 1: **Figure S1.** The breeding scheme of cKO and its littermate controls. (TIF 61 kb)

Additional file 2: Material and Methods. (DOCX 133 kb)

Additional file 3: **Figure S2.** Various measures of spatial memory in the probe tests of the Morris-water maze. (TIF 56 kb)

## Abbreviations

CaMKIIa: Ca2+/calmodulin-dependent kinase IIa
cKO: Conditional knockout
IGF: Insulin-like growth factor
NAD: Nicotinamide adenine dinucleotide
PTSD: Post-traumatic stress disorder
SIRT: Sirtuin
