# Supplementary figures and images for: Elevated contextual fear memory by SIRT6 depletion in excitatory neurons of mouse forebrain
Source: Mol Brain. 2018 Sep 6;11:49. doi: 10.1186/s13041-018-0391-6 (PMC6127998; doi:10.1186/s13041-018-0391-6)

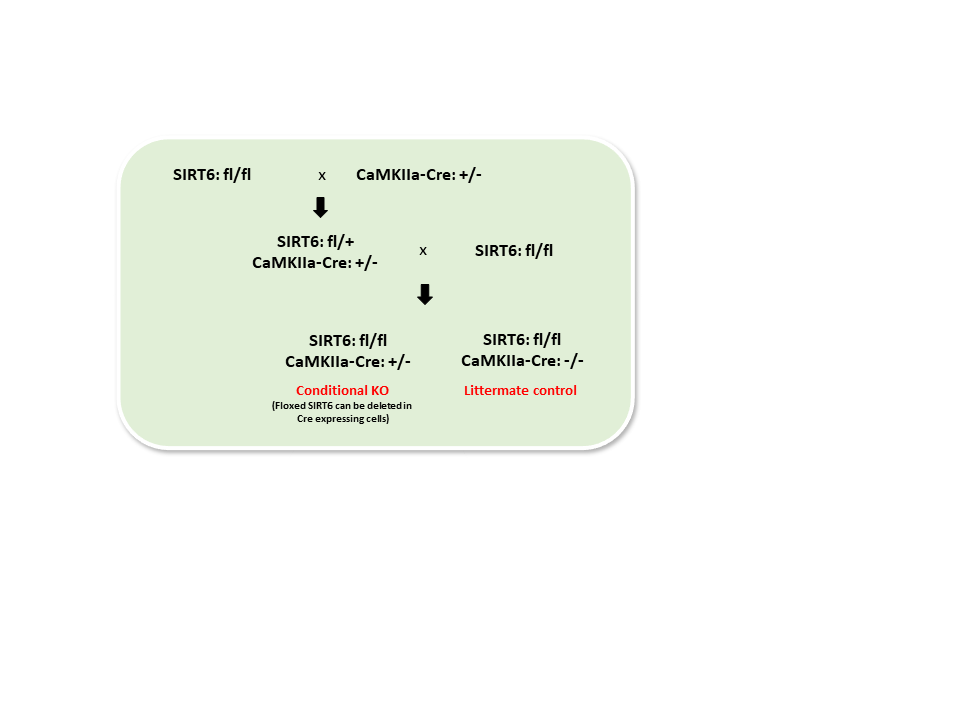

Supplement: Supplementary file 1 — Figure S1. The breeding scheme of cKO and its littermate controls. (TIF 61 kb) [file 13041_2018_391_MOESM1_ESM.tif]

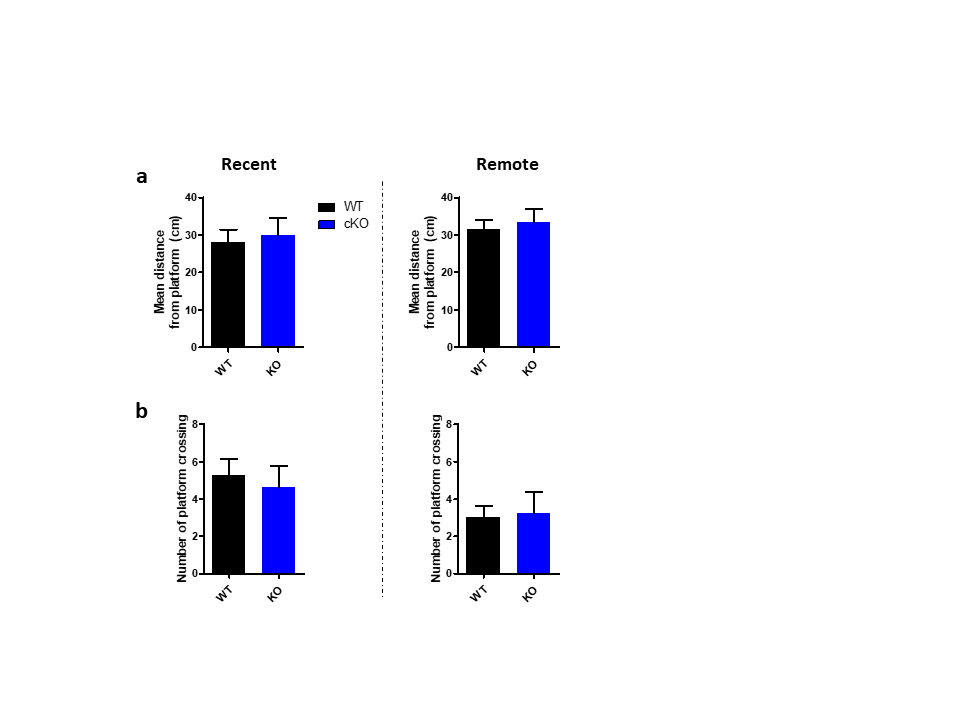

Supplement: Supplementary file 3 — Figure S2. Various measures of spatial memory in the probe tests of the Morris-water maze. (TIF 56 kb) [file 13041_2018_391_MOESM3_ESM.tif]
