# Supplementary material for: Elevated contextual fear memory by SIRT6 depletion in excitatory neurons of mouse forebrain
Source: Mol Brain. 2018 Sep 6;11:49. doi: 10.1186/s13041-018-0391-6 (PMC6127998; doi:10.1186/s13041-018-0391-6)
Supplement: Supplementary file 2 — Material and Methods. (DOCX 133 kb) [file 13041_2018_391_MOESM2_ESM.docx]

**Additional files for**

**Title:** Elevated contextual fear memory by SIRT6 depletion in excitatory neurons of mouse forebrain

**Authors:** Hyopil Kim^1^, Hyun-Seok Kim^2^, Bong-Kiun Kaang^1^

**Material and Methods**

**Animals**

The Floxed Sirt6 mouse line was procured from Dr. Hyun Seok Kim of Ewha Womans University and bred with CaMKIIa-Cre mouse line at Seoul National University. 8–20 weeks old male and female Sirt6 cKO mice and their WT littermates were used in the behavior experiments. All mice were housed under a 12-h light/dark cycle with food and water provided ad libitum. The behavioral experiment was performed during the light phase of the cycle. All procedures, which were conducted according to the policies and regulations for the care and use of laboratory animals, were approved by the Institutional Animal Care and Use Committee of Seoul National University.

**Contextual fear conditioning**

Mice were handled for 3 min for 4 consecutive days before the conditioning. After the handling, a mouse was placed in a conditioning chamber (Coulbourn) for 210 secs and two electrical foot shocks (2 secs, 0.6 mA) were delivered at 150 secs and 180 secs respectively. Freezing level was analyzed by Freeze Frame program (Coulbourn) before the first shock. After 24 hours, the mouse was returned to the chamber for 180 secs and freezing level was analyzed. The experimenters were blinded to genotype.

**Morris Water Maze (MWM) Test**

Mice were handled for 3 min for 4 consecutive days before the training. During the 5 days of training, the mice were placed in a white opaque water tank (diameter 140 cm, height 100 cm, 22–23 °C of water) in a room with multiple spatial cues. The tank was divided into 4 virtual quadrants (TQ: target quadrant, OQ: opposite quadrant, AQ1: adjacent quadrant 1, AQ2: adjacent quadrant 2), and a platform (diameter: 10 cm) was placed 1 cm below the water surface in TQ. On training days, the mice were pseudo-randomly released in one of the quadrants and trained to swim to the platform within 60 sec. If a mouse did not reach the platform within 60 s, it was guided to the platform. When a mouse reached the platform within 60 secs and stayed on the platform for more than 1 sec, it was take to the home-cage. Mice were trained with four trials per day with an intertrial interval of 1 min. 24 h after the final training trial, a probe test (recent) was performed without platform for 60 secs and their movement and position tracked with EthoVision 9.0. 28 days after the training, another probe test (remote) was performed. Time duration in each quadrant was analyzed in the probe tests. The experimenters were blinded to genotype.

**Elevated Zero Maze Test (EZM)**

Mice were taken in front of the test room for 30 min to habituate them to the environment. Then the mice were placed in the center of one of the closed arms of the maze (inner diameter: 50 cm, outer diameter: 60, consists of two open arms and two closed arms with 20-cm walls) and their movements and position were tracked for 5 min with EthoVision 9.0 (Noldus Information Technology bv, Wageningen, The Netherlands). If a mouse fell onto the floor, the mouse was excluded from the analysis. The experimenters were blinded to genotype.

**Open-Field Test (OFT)**

Mice were taken in front of the test room for 30 min to habituate them to the environment. Then the mice were placed in a square opaque white box (40 × 40 × 40 cm). Their movements in the box were tracked for 30 min with EthoVision 9.0. The experimenters were blinded to genotype.

**Supplementary Figures and Figures legends**


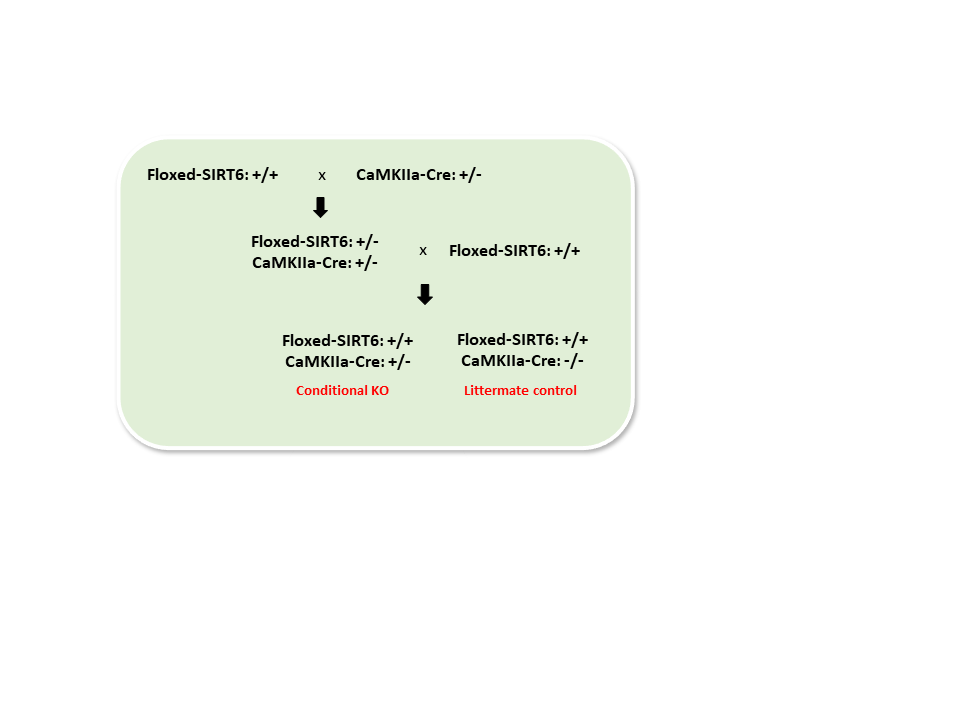


**Figure S1. The breeding scheme of cKO and its littermate controls.**

The breeding scheme of cKO and its littermate controls was represented as a figure.


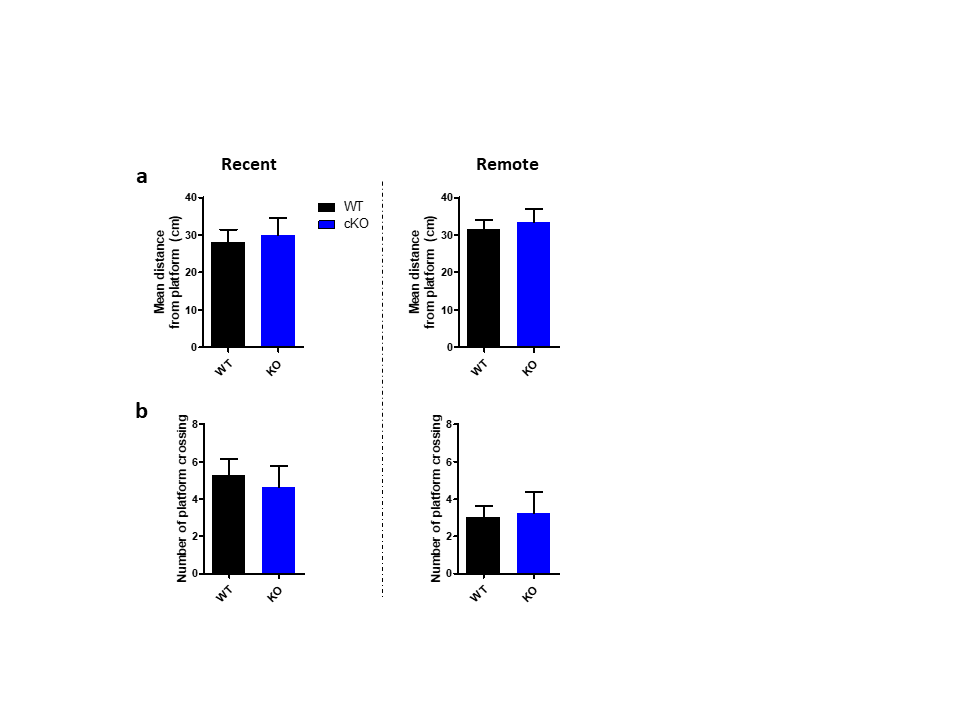


**Figure S2. Various measures of spatial memory in the probe tests of the Morris-water maze.**

(a, b) Mean distance from platform and number of platform crossing of cKO mice were comparable to those of WT controls in both recent and remote probe tests.
